# Biological Studies of New Implant Materials Based on Carbon and Polymer Carriers with Film Heterostructures Containing Noble Metals

**DOI:** 10.3390/biomedicines10092230

**Published:** 2022-09-08

**Authors:** Svetlana I. Dorovskikh, Evgeniia S. Vikulova, David S. Sergeevichev, Tatiana Ya. Guselnikova, Alexander A. Zheravin, Dmitriy A. Nasimov, Maria B. Vasilieva, Elena V. Chepeleva, Anatoly I. Saprykin, Tamara V. Basova, Natalya B. Morozova

**Affiliations:** 1 Nikolaev Institute of Inorganic Chemistry Siberian Branch of the Russian Academy of Sciences SB RAS, 3 Lavrentiev Ave., 630090 Novosibirsk, Russia; 2 «E. Meshalkin National Medical Research Center» of the Ministry of Health of the Russian Federation, 15 Rechkunovskaya Str., 630055 Novosibirsk, Russia; 3 Rzhanov Institute of Semiconductor Physics SB RAS, 13 Lavrentiev Ave., 630090 Novosibirsk, Russia; 4 Zelman Institute for the Medicine and Psychology, Novosibirsk State University, 1, Pirogov Str., 630090 Novosibirsk, Russia

**Keywords:** precious metals, chemical vapor deposition, thin films and nanoparticles, cytological studies, antibacterial studies, histological studies, carbon, polymer

## Abstract

This paper presents pioneering results on the evaluation of noble metal film hetero-structures to improve some functional characteristics of carbon-based implant materials: carbon-composite material (CCM) and carbon-fiber-reinforced polyetheretherketone (CFR-PEEK). Metal-organic chemical vapor deposition (MOCVD) was successfully applied to the deposition of Ir, Pt, and PtIr films on these carriers. A noble metal layer as thin as 1 µm provided clear X-ray imaging of 1–2.5 mm thick CFR-PEEK samples. The coated and pristine CCM and CFR-PEEK samples were further surface-modified with Au and Ag nanoparticles (NPs) through MOCVD and physical vapor deposition (PVD) processes, respectively. The composition and microstructural features, the NPs sizes, and surface concentrations were determined. In vitro biological studies included tests for cytotoxicity and antibacterial properties. A series of samples were selected for subcutaneous implantation in rats (up to 3 months) and histological studies. The bimetallic PtIr-based heterostructures showed no cytotoxicity in vitro, but were less biocompatible due to a dense two-layered fibrous capsule. AuNP heterostructures on CFR-PEEK promoted cell proliferation in vitro and exhibited a strong inhibition of bacterial growth (*p* < 0.05) and high in vitro biocompatibility, especially Au/Ir structures. AgNP heterostructures showed a more pronounced antibacterial effect, while their in vivo biocompatibility was better than that of the pristine CFR-PEEK, but worse than that of AuNP heterostructures.

## 1. Introduction

Current trends in the development of implantation in various fields of medicine, including reconstructive and oncological surgery, are associated with the creation of new biomaterials that can perform several functions, including the promotion of tissue regeneration and preventing adverse reactions and inflammatory infections. Some such materials for implants are carbon composite materials (CCMs), as well as carbon fiber reinforced polymers (e.g., carbon-fiber-reinforced polyetheretherketone (CFR-PEEK)). Unlike traditionally used metals and their alloys, these materials do not have tissue reactions and show low toxicity [1,2] which is the basis for the development of technologies for manufacturing new generation implants.

CCMs are durable porous composites of carbon fibers bonded by a carbon matrix and are characterized by structural and biomechanical compatibility, mechanical robustness, and non-toxicity. CCMs’ mechanical characteristics are as close as possible to human bones [3,4,5,6,7] and products made from them are 2.5 times lighter than similar titanium products and 5–7 times lighter than medical steel products. CCMs are less susceptible to corrosion and fatigue destruction, which are among the main causes of failure of metal endoprostheses of joints [8,9,10,11]. Thus, an increase in service life can be expected, and the need for the usually more difficult operation to replace the inserted implant can be avoided. Moreover, unlike metal plates, screws and rods, these materials do not require surgical extraction after bone fusion [12]. CCMs significantly expand the capabilities of traumatologists, orthopedists, oncologists, and neurosurgeons in the treatment of diseases of the musculoskeletal system, replacement of bone defects in the cerebral part of the skull, etc. However, appreciating the possibilities of carbon products, it should be noted that carbon is an X-ray transparent material, which makes visualization difficult and, accordingly, causes problems during fixation.

Polymer carriers are composite biomaterials based on biocompatible polymers (mainly polyetheretherketone) and carbon fibers (CFR-PEEK) [4,5,13] and are highly effective as spinal interplate implants [14,15,16]. In this case, carbon fibers act as reinforcing agents. The advantages of such materials are the possibility of varying the mechanical characteristics by changing the content of the carbon component, which allows selecting ideally compatible materials for a specific task [15,16]. However, polymers in the process of biological aging can release low-molecular weight products that can have toxic and carcinogenic effects on the human body [17]. In addition, due to the biological inertia, modification of the CFR-PEEK surface remains a serious problem. To determine the prospects for the use of such materials in orthopedics, including in oncosurgery, it is necessary to conduct more detailed studies, including clinical trials, because reliable clinical results from the study of their safety are not presented in the literature [15,16]. Similarly to metal systems, problems of peri implant infection and insufficient osteoconductivity are also observed for these materials [15,16,18,19]. In addition, polymer composite materials, as well as CCMs, are X-ray transparent and, accordingly, require labeling [15].

Infectious complications remain a common problem of these biomaterials. Therefore, the development of innovative technologies related to the design and manufacture of implants with surfaces exhibiting anti-infective, and antibacterial properties is an urgent and timely task [20,21].

Thus, in order to increase the effectiveness of new materials for implants, it is necessary that the surface modification not only be able to improve the biointegrative properties of implants (i.e., promote osteo- and histogenesis), but also provide a long-lasting antibacterial effect [22,23]. One approach to such modification is the development of methods for deposition of multifunctional biocompatible coatings based on platinum group metals and gold and silver nanoparticles on the surface of implants, which can improve their operational characteristics, namely biocompatibility and antibacterial properties. An additional function of the films based on platinum group metals is to increase the radiopacity of the coated products, which allows simultaneous marking of the implants.

Metal-organic chemical vapor deposition (MOCVD) was chosen as the deposition method. This method makes it possible to obtain noble metal coatings at low temperatures during a controlled process. In this method, volatile precursors (noble metal complexes with organic ligands) are heated in a vacuum reactor to transfer them to the gas phase and then transported in a carrier gas flow to the deposition zone, where an activated chemical reaction of decomposition of precursor vapors occurs on the heated surface of the implant with the formation of a metal coating and removal of gaseous reaction products. This method has already been successfully used and described in detail in our previous publication [24]. The method of physical vapor deposition (PVD) was used to deposit silver nanoparticles onto the film structures.

In our previous work [24], we analyzed the dependence of the biological properties of implants made of titanium and its alloys and covered with thin film structures of iridium, platinum, and gold and silver nanoparticles on the composition of these structures. 

In this work, the surface of new materials for implants, viz. CCM and CFR-PEEK, was modified with platinum group metals in combination with gold and silver nanoassemblies. The cytotoxicity, radiopacity, and antibacterial properties of the prepared film structures were for the first time studied to evaluate the effectiveness of new biomaterials. Apart from this, we conducted histological studies, as well as studies of the effects of modified surfaces on bacterial colonies, depending on their chemical composition and microrelief.

## 2. Materials and Methods

### 2.1. Synthesis and Characterization of MOCVD Precursors

Volatile complexes Ir(CO)_2_(acac), (CH_3_)_3_Pt(acac)Py and (CH_3_)_2_Au(thd), where acac = pentane-1,3-dionato(-), Py = pyridine, and thd = 2,2,6,6-trimethylheptane-3,5-dionato(-), were used as precursors for the deposition of metal coatings and nanoparticles. These compounds were synthesized by standard methods described in [25] and characterized by elemental analysis (CARLO-ERBA-11008 analyzer) and melting point (m.p.) determination (Kofler table). The detailed synthesis and identification of the gold precursor were described in our previous work [24].

Ir(CO)_2_(acac) was synthesized in the Schlenk apparatus by passing an excess of CO through a solution of 1 g (2.5 mmol) of [Ir(cod)(acac)] (97%, Sigma-Aldrich, St. Louis, MI, USA, CAS 12154-84-6) in 50 mL of hexane for 2 h. After removing the solvent in a vacuum, the product was purified by zone sublimation (10^−2^ Torr, 80–110 °C); the yield was 80% (0.69 g, 2 mmol). For IrC_7_H_7_O_4_ (mass. %) found: C, 24.3; H, 1.9, calc.: C, 24.2; H, 2.1; m.p. 145 °C (dec.) [25].

(CH_3_)_3_Pt(acac)Py was synthesized by the reaction of 1 g (2.7 mmol) of [(CH_3_)_3_PtI]_4_ (99%, Alfa-Aesar, Haverhill, MA, USA, CAS 14364-93-3) and 0.45 g (3 mmol) of K(acac) hemihydrate (97%, Alfa-Aesar, Haverhill, MA, USA, CAS 57402-46-7) in 50 mL of benzene-ethanol (1:1) solvent mixture in the presence of 0.25 mL (3 mmol) of Py (99%, Dalchem, Nizhny Novgorod, Russia, CAS 110-86-1). The product was purified by zone sublimation (10^−2^ Torr, 110 °C); the yield was 95%. For PtC_13_H_21_NO_2_ (mass. %) found: C 37.6; H 5.1; N 2.9, calc.: C 37.3; H 5.1; N 3.3; m.p.118 °C [25].

In general, all the synthesized precursors had mass fraction purities ≥ 99%.

### 2.2. Carrier Materials and Their Characterization 

Carbon composite material (further “CCM”, carbon content 99.9%, produced by NIIGrafit., Co., Ltd., Moscow, Russia) and carbon-fiber reinforced polyetheretherketone (further “CFR-PEEK”, carbon fiber content 30%, produced by Jiangsu Junhua High Performance Specialty Engineering Plastics (Peek) Products Co., Ltd., Changzhou, Jiangsu, China) were used as base carrier materials for medical implants. Both CCM discs (diameter 16–24 mm, thickness 3 mm) and CFR-PEEK discs (diameter 20–30 mm, thickness 1–2.5 mm) were degreased in isopropyl alcohol before use in MOCVD. Thermal stability under heating in an oxygen atmosphere was investigated by TGA (TG 209 F1 Iris Thermo Microbalance, NETZSCH Erich Netzsch GmbH & Co. Holding KG, Selb, Germany).

### 2.3. Deposition of Ir, Pt, PtIr Metal Films and Au, Ag Nanoparticles

Deposition of Ir, Pt, and PtIr films onto CCM and CFR-PEEK discs was performed in a vertical MOCVD reactor with cold walls [24] equipped with a Xe lamp (vacuum ultraviolet, VUV, λ = 113 nm) at reduced pressure (2 Torr). Before the deposition process, the CFR-PEEK discs were heated in the MOCVD reactor at 280 °C for 30 min under VUV radiation. The deposition conditions and the samples obtained are listed in Table 1.

Au nanoparticles were deposited by pulsed MOCVD [24] from [(CH_3_)_2_Au(thd)] on pristine CCM, CFR-PEEK and the corresponding Pt-, Ir-, and PtIr-coated carriers (Table 1). The experiments were carried out at deposition and evaporator temperatures of 320 °C and 70 °C, respectively, and the total reactor pressure of 8 Torr, using an Ar/H_2_ gas mixture (2:1) as a reagent. The experiments included 10 cycles with 2 min duration. Thus, the following samples were obtained: Au/CCM, Au/CFR-PEEK, Au/Ir/CCM, Au/Pt/CCM, Au/Ir/CFR-PEEK, Au/Pt/CFR-PEEK, and Au/PtIr/CFR-PEEK.

Ag nanoparticles were deposited by thermal physical vapor deposition (PVD) on pristine CCM, CFR-PEEK and the corresponding Pt-, Ir-, PtIr-coated carriers (Table 1). Evaporation of metallic silver (5 mg) was carried out at 1573 °C in a UVM.71-PVD installation (I = 400 mA, U = 400 B). The total pressure was 8 × 10^−7^ Torr, and the substrate temperature was 50 °C. Thus, the following samples were obtained: Ag/CCM, Ag/CFR-PEEK, Ag/Ir/CCM, Ag/Pt/CCM, Ag/Ir/CFR-PEEK, Ag/Pt/CFR-PEEK, and Ag/PtIr/CFR-PEEK.

### 2.4. X-ray Diffraction Analysis

To determine phase composition of film mono- and heterostructures, the X-ray diffraction (XRD) method was used. The diffractograms were recorded using a Shimadzu XRD-7000 diffractometer, Japan with the step of 2θ = 0.0143° (2θ range was 10–65°) and CuKα radiation (Ni filter, linear detector OneSight); the accumulation time was 5 s. The diffraction patterns were indexed using PowderCell 2.4 and WINFIT 1.2.1 programs [26]. The calculations of the unit cell parameters (UCP) of bimetallic PtIr films were performed in the PowderCell 2.4 program [27], taking into account the external standard (pristine CCM, CFR-PEEK). Coherent scattering regions (CSR) were estimated according to the Scherrer equation, taking into account the half-widths of a polycrystalline silicon standard.

### 2.5. Microscopy

Microstructural features were studied using a scanning electron microscope HITACHI UHR FE-SEM SU8200 connected with an EDX analyzer. Cross-section SEM images were obtained using a scanning electron microscope TESCAN AMBER GMH. The surface morphology of samples was studied by atomic force microscopy (AFM) in a semi-contact mode with an Ntegra Prima II (NTMDT, Moscow, Russia) microscope: length was 123 µm, width was 34 µm, force constant was 17 N/m, resonance frequency was 230 kHz. The roughness parameters were calculated using the Nova SPM software according to the standards ISO 4287-1, ISO 4287, and ASME B46.

### 2.6. X-ray Photoelectron Spectroscopy

Films’ composition was studied by X-ray photoelectron spectroscopy (XPS) using a SPECS spectrometer, Germany (Al Kα radiation, hv = 1486.74 eV, 200 W), equipped with a PHOIBOS-150-MCD-9 analyzer and a FOCUS-500 monochromator. The scanning energy step was 0.1 eV, the transmittance energy of the analyzer was 50 eV, and the standard deviation was less than 0.054 eV. The sample charge was estimated using C1s carbon spectra (284.8 eV) from natural hydrocarbon surface contaminants. The binding energy scale (E_b_) was calibrated using the positions of the peak energy levels of Au 4f_7/2_ (E_b_ = 84.0 eV) and Cu 2p_3/2_ (E_b_ = 932.67 eV). The samples were bombarded with Ar^+^ ions (2.5 keV) for 10 min in order to remove the surface layer (depth of 5 nm). The spectra were processed in the CASA program (Japan) using the Doniak-Sanjik function [28], taking into account asymmetry of peaks. The Shirley method was used to take into account the background [29].

### 2.7. Atomic Emission Spectroscopy

The gold and silver content in the studied samples were determined by inductively coupled plasma atomic emission spectroscopy (ICP-AES) using a high-resolution spectrometer iCAP 6500 (Thermo Fisher Scientific, Waltham, MA, USA). A sample solution was injected into plasma through a nebulizer (SeaSpray type) at a rate of 0.7 mL/min using a peristaltic pump. The registration was carried out at the axial observation of plasma under the following conditions, which were recommended by the manufacturer of the iCAP 6500 spectrometer: cooling argon flow = 12 L/min, secondary = 0.5 L/min, registration time = 5 s, power supplied to an ICP inductor = 1150 W. To prepare the samples, the following reagents were utilized: deionized water, which was preliminarily purified with the Direct-Q3 system (Millipore, Burlington, MA, USA) >18 MOhm/cm, concentrated HCl and HNO_3_ acids of ultrapure grades (11125-84 and 14261-77 state standard specification), high purity argon, Au standard solution (MSDS, 170216, Merck, Darmstadt, Germany), and Ag standard solution (MSDS, 119797, Merck, Darmstadt, Germany). The sample was dissolved with a mixture (3:1) of concentrated HCl and HNO_3_. An automatic pipette with variable volumes (1.00–5.00 mL, 100–1000 µL, and 10–100 µL), a polypropylene container (20 mL), and disposable plastic tubes (15 and 50 mL) were utilized to prepare the samples for investigation. The following most intense analytical lines that did not give spectral interference were used: 208.209, 242.795, 267.595 nm for Au and 328.068, 338.289 nm for Ag. To confirm the data accuracy, a spike experiment was carried out.

### 2.8. Radiopacity Study

Radiopacity of the series of Ir/CFR-PEEK and Pt/CFR-PEEK samples as a function of the thickness of a noble metal layer was studied using a Toshiba Aquilion PRIME magnetic resonance (MR) tomograph at 120 keV, 200 mA.

### 2.9. Biological Studies

#### 2.9.1. Cytotoxicity Studies of Samples

To study the cytotoxicity of the samples L-929 fibroblasts (ATCC number: CCL-1) obtained from the cell cultures collection (SRC VB “Vector”, Koltsovo, Russia) were used. The cells were cultured in F12/DMEM medium (Thermo Fisher Scientific, Waltham, MA, USA) with the addition of 10% fetal bovine serum (Thermo Fisher Scientific, Waltham, MA, USA), 100 U/mL penicillin (Thermo Fisher Scientific, Waltham, MA, USA), 100 U/mL streptomycin (ThermoFisher Scientific, USA) and 2 mmol/L L-glutamine (Thermo Fisher Scientific, Waltham, MA, USA) in a CO_2_ incubator. Cytotoxicity of experimental samples against L-929 cells was determined using the XTT test by the indirect contact method [30]. 

For the preparation of the extracts, samples were incubated in F12/DMEM medium for 72 h at 37 °C at a ratio of 1 mL of medium per 0.1 g of sample. L-929 cells were seeded on 96-well plates at a concentration of 15,000 per well in a culture medium and incubated for 24 h for attachment. Then, the medium was withdrawn, and 100 μL of the obtained extracts were added to each well. L-929 cells in a culture medium were used as a control. There were three samples in each group. XTT test was performed after 24 and 48 h. The viability of L-929 cells was established using an iMark Microplate Absorbance Reader (Bio-Rad, Hercules, CA, USA) and an XTT cell proliferation kit (Applichem, Darmstadt, Germany) in accordance with the recommendations of the manufacturers. The results were given as a percentage of the control value.

#### 2.9.2. Investigation of Antibacterial Properties of Samples

Pure cultures of *Str. pyogenes*, *S. aureus*, *S. epidermidis*, *P. aeruginosa*, and *Ent. Faecium* were used for the study of antibacterial activity using the method adapted from Koller M. et al. [31].

**Figure 1:**
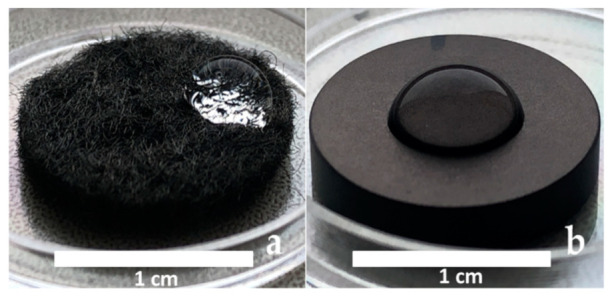
CCM (**a**) and CFR-PEEK (**b**) samples with 50 µL bacterial suspension.

Before starting, standard solutions containing 2 × 10^6^ CFU/mL each were prepared in RPMI-1640 medium (Thermo Fisher Scientific, USA) with the addition of 0.3 g/L L-glutamine and 2 g/L sodium bicarbonate (Thermo Fisher Scientific, USA), and samples were placed in wells of a 6-well plate. Fifty microliters of the bacterial suspension was applied as a drop to each test sample and incubated in a humid thermostat at 37 °C (Figure 1). The next day, a drop of bacterial suspension from the sample was transferred to a Petri dish with appropriate culture medium and cultured further for 24 h to evaluate bacterial growth. Bacterial colonies were stained with Hoechst 33342 fluorescent dye (Thermo Fisher Scientific, USA) and were counted using an Axioskop 40FL microscope (Carl Zeiss, Jena, Germany). Measurements in each experimental group were performed in three repetitions.

#### 2.9.3. In Vivo Biocompatibility and Histological Studies

The Local Ethics Committee of the E. Meshalkin National Medical Research Center of the Ministry of Health of the Russian Federation approved all in vivo biocompatibility and histological studies. All parts of the protocol were implemented according to the recommendations on the proper use and care of laboratory animals (European Communities Council Directive 86/609/CEE) and the principles of the Declaration of Helsinki). Fifteen pairs of the prepared samples were implanted subcutaneously in Wistar rats (weight of 100–120 g, n = 15). Rats were anesthetized using intraperitoneal injection (30 mg/kg Zoletil-100, Virbac, Carros, France). The hair on the animals’ backs was shaved off, and after that, the skin was treated with betadine. Each animal had two experimental specimens placed in pre-formed subcutaneous pockets on either side of the back. The incision of skin was sutured with Prolen 4/0 (B.Braun, Bethlehem, PA, USA) and treated with betadine. Implanted samples together with the surrounding tissues were removed after 1 and 3 months for histological examination of post-implantation inflammatory reactions. Six-micrometer sections of formalin-fixed paraffin specimens were prepared using an HM340 microtome (Microm, Walldorf, Germany) and stained with hematoxylin-eosin. An Axioskop 40FL microscope (Carl Zeiss, Germany) equipped with AxioVision v. 4.7 software was used for the morphological studies. The following scheme was used for scoring: 0—no changes, 1—mild changes, 2—moderate changes, and 3—severe changes.

#### 2.9.4. Statistical Analysis

Data were presented as average values ± standard deviations (SD). The statistical analysis and significance were evaluated using Student’s *t* test. The results of all analyses were processed using the Statistica 13 software (TIBCO Software, Palo Alto, CA, USA). The difference between control and treated samples was considered significant at *p* < 0.05.

## 3. Results and Discussion

### 3.1. CCM and CFR-PEEK Carriers

Before the deposition of Ir-, Pt-, and PtIr- films and the corresponding heterostructures, the composition and microstructure of CCM and CFR-PEEK carriers were studied, and the temperature stability ranges of the CCM and CFR-PEEK materials were determined.

**Figure 2:**
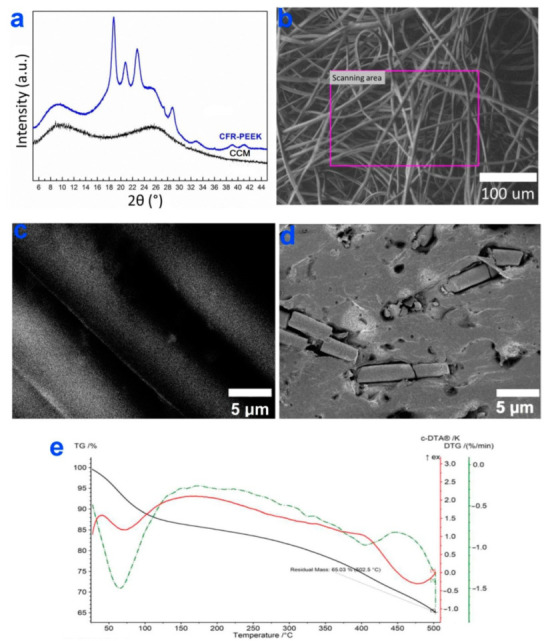
XRD patters of CCM and CFR-PEEK (**a**), SEM images of CCM (**b**,**c**) and CFR-PEEK (**d**), TG-DTA curves of CCM (**e**).

The analysis of XRD patterns of CCM and CFR-PEEK (Figure 2a) confirmed the presence of a graphite-like carbon (2θ = 9.4(110), 25.3(111)) in both samples [32]. In the case of CFR-PEEK, the main reflexes at 2θ = 18.84(110), 20.88(111), 22.92(200), 28.80°(211) belonging to PEEK polymer were also visualized [33]. From SEM data, the surfaces of CCM samples were formed by compressed graphite filaments (average wire diameter ~5 µm) (Figure 2b,c). In contrast to CCM, the surfaces of CFR-PEEK discs were not porous and consisted of smoothed areas of polymer with an average roughness of 55 nm and local inclusions of graphite-like tubes (Figure 2d). 

The study of the thermal stability of the CCM by the TGA method showed that its mass loss occurred gradually in two stages of heating in an oxidizing atmosphere. The first stage (50–100 °C, residue 87%) was associated with the release of sorbed water. Further, a slight loss of the sample mass was observed at 100–320 °C (80% residue), followed by a monotonous loss of sample mass at 330 °C due to its oxidation (Figure 2e). According to the manufacturer, vitrification and degradation of CFR-PEEK occurred above 330 °C.

Thus, in MOCVD processes, the deposition temperatures of noble metal coatings and nanoparticles on CCM and CFR-PEEK carriers should be below 330 °C, which required the selection of the special volatile metal precursors with certain characteristics.

### 3.2. Deposition and Characterization of Ir, Pt, and PtIr Films

Taking into account the data on thermal stability of CCM and CFR-PEEK carriers, the precursors Ir(CO)_2_(acac), (CH_3_)_3_Pt(acac)Py, and (CH_3_)_2_Au(thd), characterized by high volatility combined with relatively low vapor stability, were chosen for MOCVD experiments: according to high-temperature mass spectrometry, their threshold decomposition temperatures lie in the range of 200–250 °C [25].

The combination of the precursors Ir(CO)_2_(acac) and (CH_3_)_3_Pt(acac)Py was used for the co-deposition of bimetallic PtIr coatings. The ratio of metals in the PtIr samples was set as the ratio of partial vapor pressures of precursors (1:1). The experimental MOCVD parameters were selected based on our previous report [24] to deposit metal films with a thickness of more than 1 micron. The samples are listed in Table 1.

To study the effect of film thickness on the characteristics of radiopacity, the duration of deposition processes of noble metal coatings on CFR-PEEK varied from 2 to 12 h (Table 1).

#### 3.2.1. Ir Films

**Figure 3:**
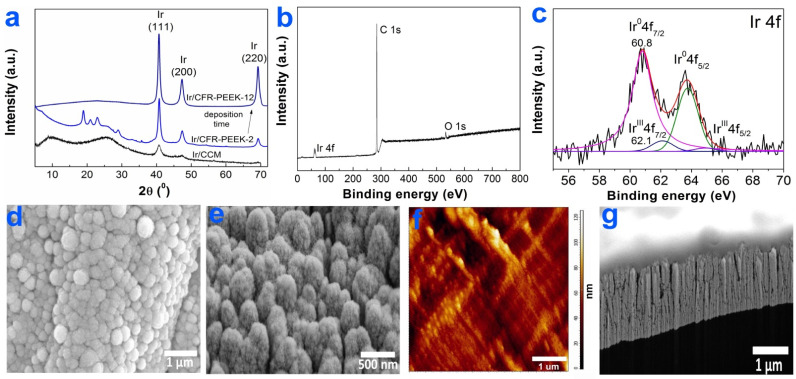
Characterization of Ir films: XRD patterns of Ir/CCM, Ir/CFR-PEEK-2, and Ir/CFR-PEEK-12 (**a**), XPS spectra of Ir/CCM (**b**), fitting of Ir4f spectra (**c**), SEM micrographs of the surfaces of Ir/CCM (**d**) and Ir/CFR-PEEK (**e**), AFM micrograph of Ir/CFR-PEEK (**f**), FIB-SEM cross-section of Ir/CFR-PEEK (**g**).

According to the XRD data (Figure 3a), the main crystal phase recorded in the Ir/CCM and Ir/CFR-PEEK samples was FCC-Ir (2θ = 40.8(111), 46.7(200), 68.8(220), JCPDS Card No.: 000-46-1044). The reflexes observed on the XRD patterns of the samples in the 2θ region of 5–35° could be assigned to CCM or CFR-PEEK materials (Figure 3a). Iridium films on CCM and CFR-PEEK carriers consisted of chaotically oriented crystallites with close average sizes of 8(2) and 11(2) nm, respectively. An increase in the deposition time of Ir/CFR-PEEK samples practically did not change the crystallite size but caused the appearance of a texture in the [110] direction (Figure 3a).

The chemical composition of Ir films was studied by XPS using an Ir/CCM sample as an example. Along with Ir4f peaks, the intense C1s and O1s peaks were observed in its XPS spectrum (Figure 3b). On the surface of Ir/CCM, Ir is present mainly in the metallic state (4f_7/2_ = 60.8 eV) with a small content of Ir^III^O_x_ phase (4f_7/2_ = 62.1 eV) (Figure 3c). In the O1s spectrum (Figure S1a), oxygen was present mainly in the adsorbed state as part of the O-H groups (533.8 eV) with a small amount of Ir-O (531.3 eV). Since the Ir^III^Ox phase was absent on the XRD patterns of Ir/CCM and other samples, it can be assumed that it was amorphous or its content was low. In the C1s spectrum, only one carbon state at 284.5 eV(C-C groups) was distinguished (Figure S1b). The C-C groups can be attributed to the CCM material or to surface contaminants (products of precursor vapor decomposition).

According to SEM data, the surfaces of Ir/CCM and Ir/CFR-PEEK samples were formed by small nanocrystallites combined into fractal-like agglomerates (Figure 3d,e). The sizes of the agglomerates varied depending on the carrier material: they were 250–800 nm for Ir/CCM samples, while in the case of Ir/CFR-PEEK samples, they were 300–700 nm (in the carbon fiber region) or 100–400 nm (the polymer region, Figure 3e). The average roughness of the Ir/CFR-PEEK sample was slightly higher than that of the bare CFR-PEEK: 68 nm vs. 55 nm (Figure 3f). FIB-SEM cross-section of the Ir film on CFR-PEEK showed a characteristic columnar dendritic microstructure [34] of the metallic layer (Figure 3g). When deposited for 4 h, the thickness of Ir films on CFR-PEEK discs reached 1.1 μm (Figure 3g).

#### 3.2.2. Pt Films

**Figure 4:**
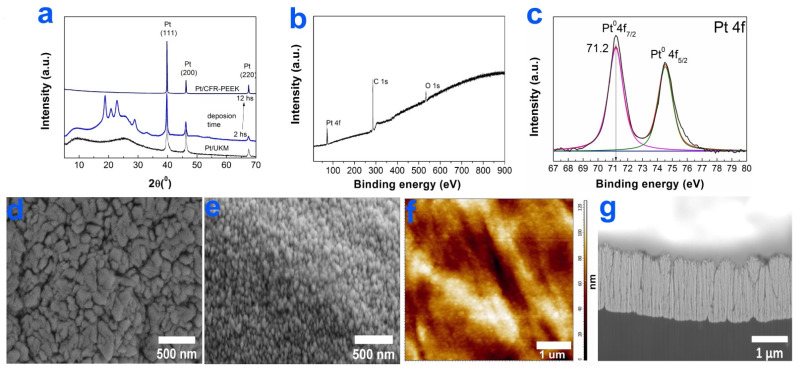
Characterization of Pt films: XRD patterns of Pt/CCM, Pt/CFR-PEEK-2, and Pt/CFR-PEEK-12 (**a**), XPS spectra of Pt/CFR-PEEK (**b**), fitting of Pt4f spectra (**c**), SEM micrographs of the surface of Pt/CCM (**d**) and Pt/CFR-PEEK (**e**), AFM micrograph of Pt/CFR-PEEK (**f**), and FIB-SEM cross-section micrograph of Pt/CFR-PEEK (**g**).

According to the XRD data, the main crystal phases recorded in the Pt/CCM and Pt/CFR-PEEK samples were FCC-Pt (2θ = 39.75(111), 46.37(200), 67.55°(220), Card No.: 010-87-0647), graphite-like carbon and/or PEEK polymer (Figure 4a). The Pt/CCM sample consisted of randomly oriented Pt crystallites with an average size of 40 nm. It was shown that in the case of Pt/CFR-PEEK samples, an increase in the process time from 2 to 12 h led to the disappearance of the CFR-PEEK reflexes on the XRD patterns (Figure 4a). Thus, the coating thickness increased significantly, while the average crystallite sizes changed from 27(3) to 34(3) nm. At the same time, an increase in the duration of the deposition process for Pt/CFR-PEEK samples promoted the growth of (111) -oriented Pt film.

The chemical composition of Pt coatings was studied by XPS using a Pt/CFR-PEEK sample as an example. Along with Pt4f peaks, the intense C1s and O1s peaks (Figure 4b) were observed in its XPS spectrum. On the surface of Pt/CFR-PEEK, platinum existed only in the metallic state (4f_7/2_ = 71.2 eV) (Figure 4c). There were three carbon states at 284.6, 286.2, and 287.7 belonging to the organic C-C, C-O, and C=O fragments, respectively, in C1s spectrum (Figure S2a). In the O1s spectrum, oxygen was present in two states associated with the organic C-O (532.3 eV) and C=O (533.5 eV) groups (Figure S2b). These fragments can be attributed to CFR-PEEK material [35] or to surface contaminants (products of precursor vapor decomposition).

According to the SEM data, the surface of Pt coatings deposited on CCM discs and in CFR-PEEK carbon tube areas was formed by elongated agglomerates whose sizes varied from 200 to 500 nm (Figure 4d). The surface of Pt films on CFR-PEEK in the PEEK polymer region was formed by small (up to 150 nm) particles tightly adhering to each other (Figure 4e). 

The studied Pt/CFR-PEEK sample has a roughness of 62 nm (Figure 4f), which was slightly less than that of Ir/CFR-PEEK. The Pt/CFR-PEEK films have a characteristic columnar structure [36]. The thickness of Pt films on CFR-PEEK discs reached ~1.4 μm (Figure 4g) when the deposition time was 4 h.

#### 3.2.3. PtIr Films

**Figure 5:**
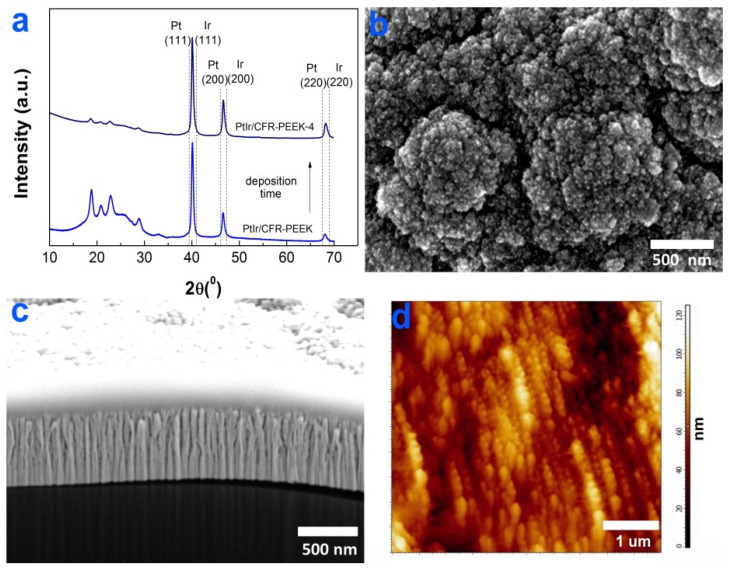
Characterization of PtIr films: XRD patterns of PtIr/CFR-PEEK, PtIr/CFR-PEEK-4 (**a**), SEM surface micrograph of PtIr/CFR-PEEK (**b**), AFM micrograph of PtIr/CFR-PEEK-4 (**c**), and FIB-SEM cross-section micrograph of PtIr/CFR-PEEK (**d**).

From XRD data, the PtIr/CFR-PEEK and PtIr/CFR-PEEK-4 samples were formed by a solid solution based on FCC metals (Figure 5a), and the reflexes related to CFR-PEEK were observed in the 2θ region of 5–35°. The films were formed by randomly oriented crystallites with a size of 20(2) nm. According to the Vegard model [37], the ratio of metals in the PtIr/CFR-PEEK samples was close to the given one: 1:1 (a = 3.870Å). According to EDX data, the Pt:Ir ratio was 40:60 (at. %).

Similarly to the Ir/CFR-PEEK samples, the surface of PtIr/CFR-PEEK was formed by fractal-like agglomerates with dimensions of 300–900 nm (Figure 5b). Due to the similar surface morphology, the roughness of the PtIr coatings was close to the Ir and Pt layers: 71 ± 2 nm (Figure 5c). An increase in the deposition time from 2 to 4 h led to the expansion of the surface agglomerates. The thickness increased approximately twice: from 0.77 ± 0.04 to 1.4 ± 0.07 μm. The PtIr coatings had a columnar microstructure that was “intermediate” compared to the Pt and Ir layers (Figure 5d). 

### 3.3. Radiopacity Study of Ir- and Pt-Coated CFR-PEEK Samples

Radiopacity was evaluated for Ir and Pt coating samples on CFR-PEEK carriers because the CFR-PEEK material contained both carbon and polymeric components of the carrier biomaterials studied in this work. The thickness of noble metal coating on CFR-PEEK was calculated as the ratio of the weight of the deposited metal to its density, normalized to the geometric surface area (Table 2). The applicability of this thickness estimation method was confirmed using a Pt/CFR-PEEK sample as an example by comparing its thickness measured from the FIB-SEM data with the calculated one.

**Figure 6:**
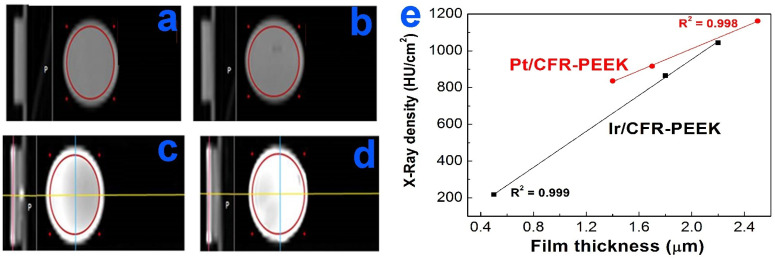
Magnetic resonance tomographic images of bare CFR-PEEK disc (**a**), Ir/CFR-PEEK-2 (**b**), Ir/CFR-PEEK-8 (**c**), and Ir/CFR-PEEK-12 (**d**) and dependences of X-ray density of samples on the Ir and Pt film thicknesses (**e**).

The X-ray density of bare CFR-PEEK was low and almost independent of the sample thickness (180–200 HU/cm^2^ for 1.0–2.5 mm) (Table 2). Most of the samples with noble metal films were characterized by high X-ray density (up to 1000–1160 HU/cm^2^). In the cross-sectional images, the coatings (Ir, Pt) and the carrier (CFR-PEEK) were clearly separated (Figure 6); for the Pt/CFR-PEEK samples, the peripheral (uncoated) areas on the surface at the CFR-PEEK fixation sites were also visualized. Thus, the main contribution to the radiopacity of the samples was from the noble metals, despite their low weight content: 7–11% in the case of Pt and 0.3–3% in the case of Ir (according to the EDX data). The X-ray density of the samples increased with coating thickness, and the dependences were close to linear (Figure 6e). It was shown that when the Ir coating thickness was equal to 0.5 μm, the sample contrast was comparable to the carrier (217 HU/cm^2^) (Figure 6).

Thus, deposition of Ir or Pt coatings with a thickness of 1–1.5 µm on CFR-PEEK ensures the achievement of a radiopaque response. These coatings can be used as markers to improve implant visualization.

### 3.4. Ir, Pt and PtIr Coatings Modified with Au Nanoparticles

AuNPs were deposited under the same experimental conditions onto CCM and CFR-PEEK discs without and with Ir, Pt, and PtIr coatings. The obtained samples were labeled as Au/CCM, Au/CFR-PEEK, Au/Ir/CCM, Au/Pt/CCM, Au/Ir/CFR-PEEK, Au/Pt/CFR-PEEK and Au/PtIr/CFR-PEEK, respectively.

**Figure 7:**
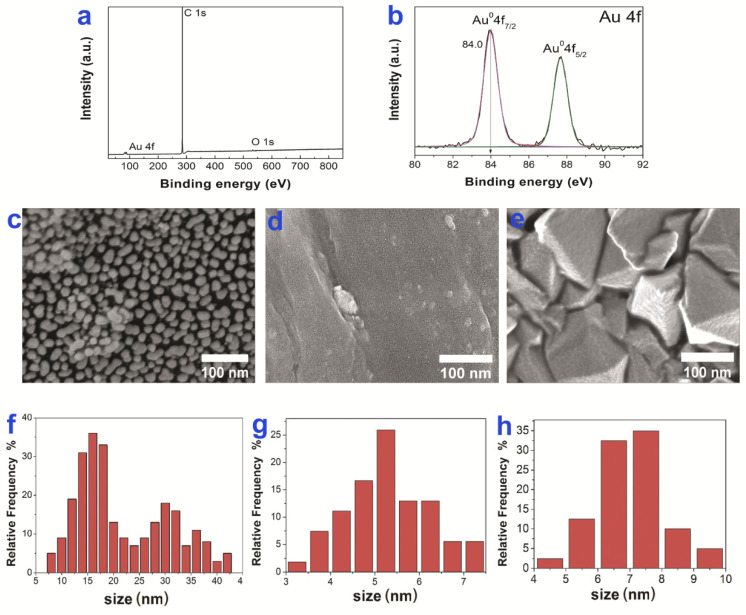
XPS spectra of Au/CCM with fitting of C1s, O1s (**a**), fitting of Au4f spectra (**b**), SEM images of Au/CCM (**c**), Au/CFR-PEEK (**d**) and Au/Pt/CFR-PEEK (**e**) with AuNPs size distributions Au/CCM (**f**), Au/CFR-PEEK (**g**) and Au/Pt/CFR-PEEK (**h**).

The gold surface concentration in the obtained samples varied within a narrow range of 10–11 μg/cm^2^. No reflexes related to the metallic gold phase were observed on XRD patterns of the above-mentioned samples due to the low content of gold in them. From XPS data presented on the example of Au/CCM sample (Figure 7a,b), the gold on the sample surface was in the metallic state (4f_7/2_ = 84.0 eV).

On the top of CCM surface, AuNPs were combined into nanoclusters with sizes of 10–42 nm (Figure 7c); smaller particles with sizes of 15–25 nm were formed in the hollows of the CCM disc. A similar gradient in AuNPs size was also observed in the case of Au/Ir/CCM and Au/Pt/CCM. The sizes of nanoclusters on the surfaces of Au/Ir/CCM and Au/Pt/CCM samples were from 30 to 50 nm.

The AuNPs (sizes 3–7 nm) with a unimodal size distribution were formed on CFR-PEEK carriers (Figure 7d). The sizes of AuNPs on the Au/Ir/CFR-PEEK, Au/Pt/CFR-PEEK, and Au/PtIr/CFR-PEEK surfaces were 6–9 nm. A typical micrograph of Au/Pt/CFR-PEEK is shown in Figure 7e. 

Probably the reasons for the morphological differences between AuNPs formed on CCM and CFR-PEEK carriers were the features of the carrier surfaces as well as the differences in the thermal conductivity of these materials.

### 3.5. Ir, Pt, and PtIr Coatings Modified with Ag Nanoparticles

AgNPs were deposited by PVD under the same experimental conditions onto CCM and CFR-PEEK discs without/with Ir, Pt, and PtIr films. The prepared samples were labeled as Ag/CCM, Ag/CFR-PEEK, Ag/Ir/CCM, Ag/Pt/CCM, Ag/Ir/CFR-PEEK, Ag/Pt/CFR-PEEK and Ag/PtIr/CFR-PEEK, respectively. 

**Figure 8:**
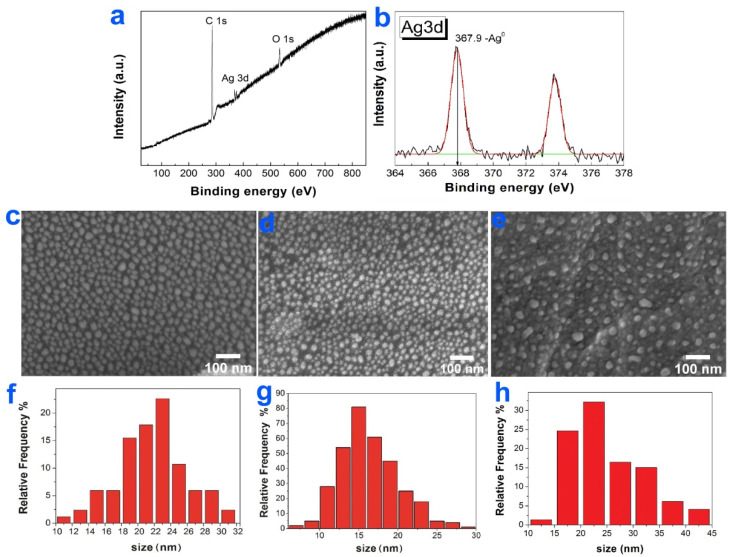
XPS spectra of Ag/CCM with fitting of C1s, O1s (**a**), fitting of Ag3d spectra (**b**), SEM images of Ag/CCM (**c**), Ag/CFR-PEEK (**d**) and Ag/Pt/CFR-PEEK (**e**) with AgNP size distributions Ag/CCM (**f**), Ag/CFR-PEEK (**g**) and Ag/Pt/CFR-PEEK (**h**).

The concentration of silver on the surface of the obtained samples varied in the range of 4–6 μg/cm^2^. Due to such a low content of silver, the corresponding peaks in the XRD patterns of the samples were not visible. At the same time, according to the XPS data presented for the Ag/CCM sample as an example (Figure 8a,b), silver on the sample surface was in a metallic state (3d_5/2_ = 367.9 eV). 

AgNPs with a unimodal distribution with sizes 18–24 and 13–20 nm, respectively, were formed on the surface of CCM and CFR-PEEK discs (Figure 8c,d). On the surface of Ir, Pt and PtIr films, AgNPs were enlarged and merged into clusters as large as 200 nm. In particular, AgNPs with sizes of 15–25 nm and Ag clusters (30–45 nm) were formed on the surface of Ag/PtIr/CFR-PEEK (Figure 8e).

### 3.6. Cytotoxic Activity

The used indirect contact method consisted of cell incubation with extracts obtained from the samples under study, followed by an assessment of cell viability. L-929 fibroblasts were the tested cell line here. 

**Figure 9:**
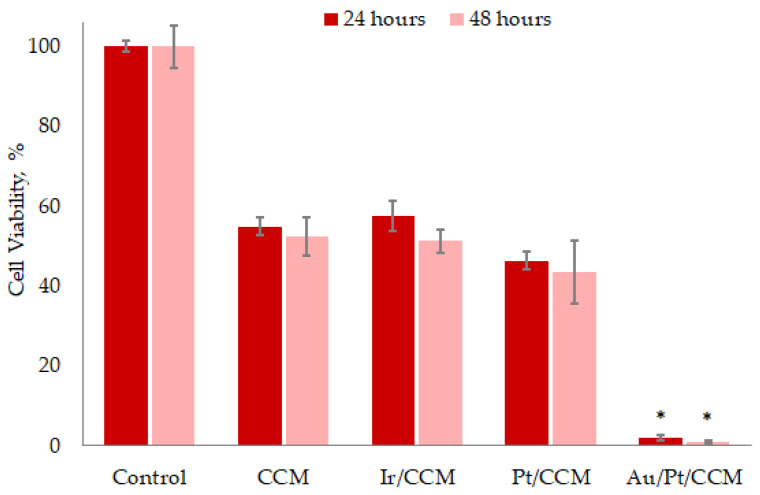
L-929 cell viability after 24 and 48 h of cultivation with extracts of pristine and CCM samples coated with different noble metals. * *p* < 0.001 (compared to the pristine CCM group).

The results for coated CCM discs compared with a pristine carrier are shown in Figure 9. For the CCM group, cell viability was just over 50%, indicating a high intrinsic cytotoxicity of this pristine material. A possible reason may be the presence of toxic compounds left after the production step and released into the biological solution. There seemed to be no time dependence of this cytotoxic effect, since the cell viability did not change after 24 h. The reason for these observations on “limited cytotoxicity” may be that the toxicant contained in the extract was inactivated by the cells. Another scenario is also possible, in which this toxicant acted permanently on all cells, reducing the total activity of mitochondrial dehydrogenases. As a result, the cells remained alive, but the efficiency of cleavage of the XTT tetrazolium yellow salt decreased with the formation of a water-soluble orange dye, which was determined calorimetrically [38].

Surface modification with noble metal films reliably did not change the pattern of cell viability: over 50% for Ir/CCM groups and over 40% for Pt/CCM groups (Figure 9). The developed microstructure of the films (columnar dendritic for Ir and columnar for Pt, Section 3.2.1 and Section 3.2.2) did not prevent the penetration of the solution to the carrier and thus did not interfere with the release of carrier toxic agents. This has previously been shown for the nickel release from Ir-coated TiNi plates [24]. It should be noted that the toxics in the CCM were quite stable, because they were not eliminated under the thermal oxidative treatment during the MOCVD experiment. A possible way to prevent the observed toxic effect is to form a thin protective noble metal sublayer of compact microstructure.

At the same time, the addition of AuNPs dramatically enhanced cytotoxicity: in the Au/Pt/CCM group, there were almost no viable cells (Figure 9). There may be several reasons for this observation. In particular, AuNPs could synergistically enhance the above described actions of the toxicant released by CCM. The ability of AuNPs to exhibit amplification or even change the biological effect of different compounds has been shown repeatedly. A prime example was the appearance of a synthetic toxic effect when these were added to biocompatible industrial surfactants [39]. Alternatively, it could be an independent toxic action of the AuNPs activating the WNT signaling pathway, developing oxidative stress and cell death [40,41,42] that adds to the effect produced by the CCM. Moreover, a synergistic/galvanic effect of the Au-Pt pair that increases biological action including toxicity [43] is also possible here. In this case, the absence of such an effect for the counterpart of the Au/Pt heterostructure deposited on the CFR-PEEK (see below) may be due to the carrier geometry. In fact, the filamentous structure of the CCM disc significantly increases the active surface area in comparison with the CFR-PEEK.

**Figure 10:**
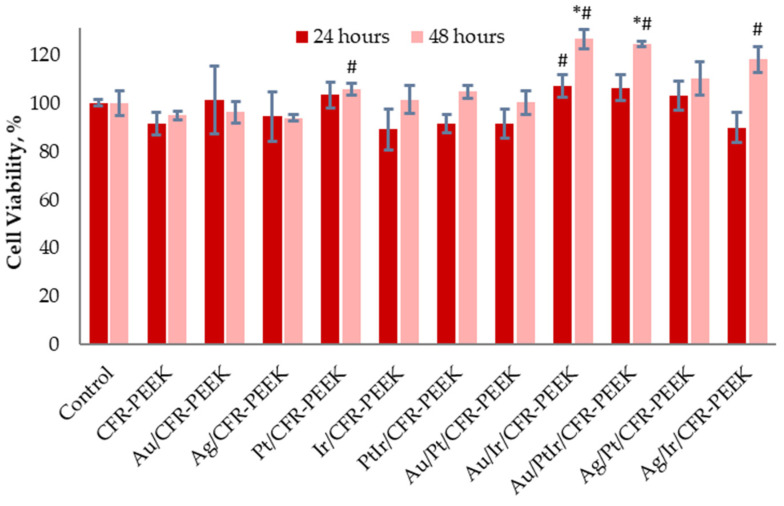
L-929 cell viability after 24 and 48 h of cultivation with extracts of pristine and coated CFR-PEEK samples with different noble metals. * *p* < 0.05 (as compared to the control group). # *p* < 0.05 (as compared to the pristine CFR-PEEK group).

As for the coated and pristine CFR-PEEK discs (Figure 10), the cell viability in all the studied sample groups was more than 80%, which indicated the safety of these implant materials. Moreover, Ir- and PtIr-coated samples, including those modified with AuNPs or AgNPs, showed reliably increased cell proliferation after 48 h. It should be noted that Ir and PtIr films have the most developed surface morphology, and, accordingly, the samples were characterized by the highest roughness values (Section 3.2.1 and Section 3.2.3). The surface characteristics are known to be of great importance for fibroblast cell growth [44]. In particular, the high roughness has a positive effect on the efficiency of growth in the number of cells and an increase in mitochondrial respiration [45]. This may be the reason for the observed increase in fibroblast viability (>100%) in the XTT test for the discussed sample groups.

The proliferation of fibroblasts and their differentiation into fibrocytes and the synthesis of the extracellular matrix by these cells reflects the course of the processes of connective tissue formation. The fine regulation of these processes depends on the microenvironment, in the formation of which a foreign body (implanted material) can also participate. The following histological studies of the structure of the fibrous tissue surrounding the noble metal-coated experimental samples will show correlations with these in vitro results.

### 3.7. Antibacterial Activity

**Figure 11:**
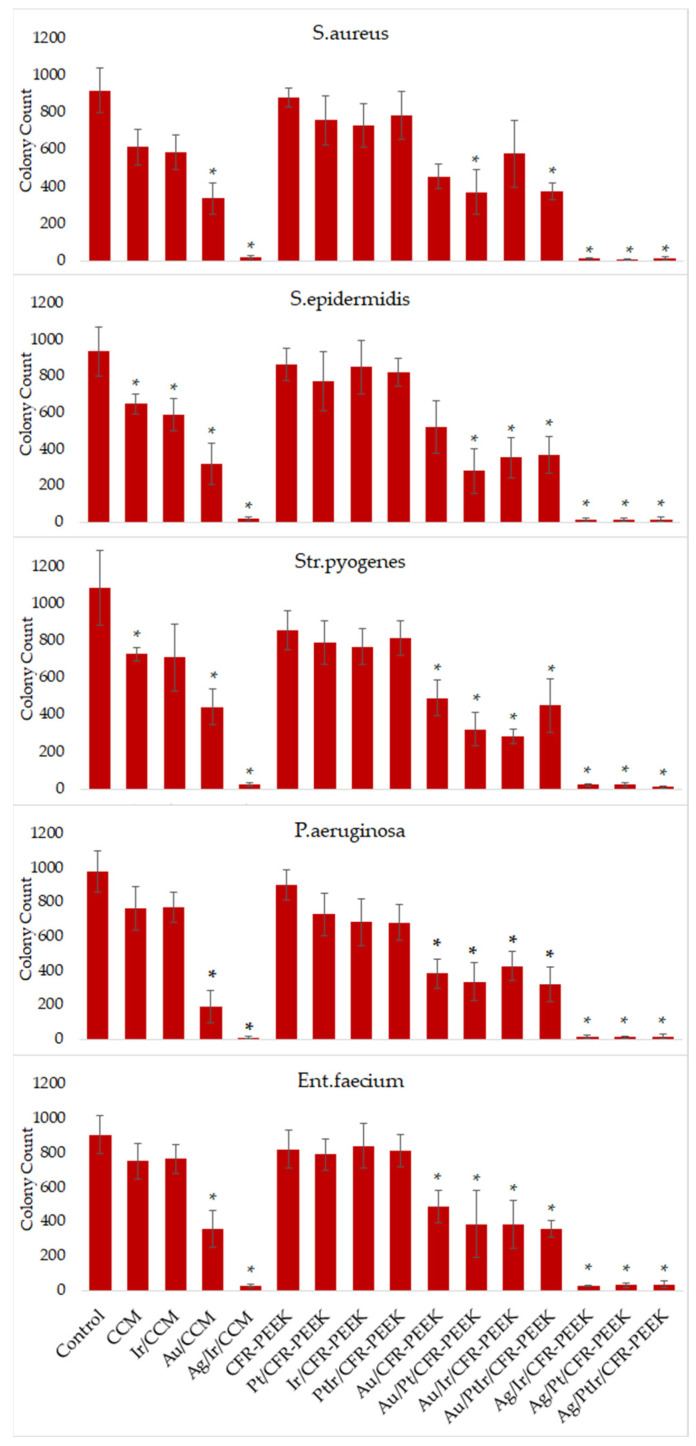
Profile300 of antibacterial activity of novel noble metal-coated materials against *S. aureus*, *S. epidermidis*, *Str. pyogenes*, *P. aeruginosa* and *Ent. faecium*. * *p* < 0.05 (as compared to the control).

Studies of the antibacterial activity of the pristine and coated implant materials showed no pronounced features of the observed effect depending on the bacterial culture (*Str. pyogenes*, *S. aureus*, *S. epidermidis*, *P. aeruginosa* or *Ent. Faecium*). In general, the pictures of the antibacterial action of the samples were similar for all strains (Figure 11). 

**Figure 12:**
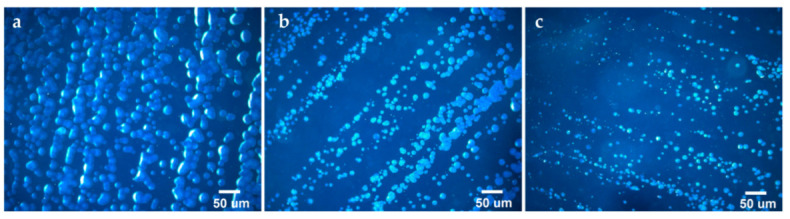
Fluorescence optical microscope images of *S. aureus*, illustrating the different degrees of bacterial colony growth: (**a**) total growth; (**b**) slightly stunted growth; (**c**) strongly stunted growth.

In quantitative terms, the control group was characterized by the number of counted colonies = 900–1000, and all the sample groups could be divided into four series:

- Total growth of bacterial colonies (colony counts ≥ 700, Figure 12a): control, CFR-PEEK, Pt/CFR-PEEK, Ir/CFR-PEEK, PtIr/CFR-PEEK;
- Slightly stunted growth (colony counts = 500–700, Figure 12b): CCM, Ir/CCM;
- Strongly stunted growth (colony counts = 300–500, Figure 12c): Au/CCM, Au/CFR-PEEK, Au/Ir/CFR-PEEK, Au/Pt/CFR-PEEK, Au/PtIr/CFR-PEEK;
- No growth (colony counts = 0–300): Ag/Ir/CCM, Ag/Ir/CFR-PEEK, Ag/Pt/CFR-PEEK, Ag/PtIr/CFR-PEEK.

Thus, a pristine CCM disc, in contrast to CFR-PEEK, exhibited some antibacterial effect. This can be associated with the same reasons explaining the increased cytotoxicity of the used CCM (Section 3.6). The application of noble metal films does not affect the observed characteristics of the original carriers: groups of coated samples belonged to the same series as uncoated ones. Conversely, the surface modification with NPs contributed a noticeable antibacterial effect.

In particular, both AuNP-modified carriers (Au/CCM, Au/CFR-PEEK) and AuNP heterostructures (Au/M/CFR-PEEK, M = Ir, Pt, PtIr) exhibited a strong inhibition of bacterial growth (*p* < 0.05). When analyzing all bacterial cultures, these samples were calculated to lead to a decrease in the number of colonies by an average of three times. The inhibition of bacterial growth by gold nanoparticles can be explained by several mechanisms. On the one hand, gold cations can interact with phospholipid components and reduce the dipole potential of the bacterial wall, which leads to a change in the total membrane charge and its local destruction, an increase in permeability and the formation of free radicals. On the other hand, intracellular proteins are original targets for metal toxicity due to their multiple amino acid binding sites, mainly consisting of reduced thiols from cysteine side chains, carboxy groups of aspartates and glutamates, and the highly reactive primary amines of lysine side chains. Once bound, the metal ions catalyze the oxidation of sensitive amino acids, disrupting protein function, reducing protein stability and marking the protein for elimination [46,47,48]. 

At the same time, the AgNP-containing samples led to the decrease by an average of 62 times. Thus, the presence of AgNPs provides the maximum effect despite a two-times smaller surface concentration of silver compared to gold. The achieved complete suppression of the growth of bacterial colonies in all cases did not allow tracking the effect of the noble metal layer on the effectiveness of the silver action, which would correspond to the sacrificial anode principle in such film heterostructures [24,31]. 

It should be noted that, unlike similar mono- and heterostructures deposited on titanium discs [24] where only Ag-containing samples showed antibacterial activity, AuNP-modified ones obtained here also exhibited the desired effect. It is known that the antibacterial effect increases as the AuNP size decreases, which is due to an increase in the efficiency of nanoperforation of the bacterial wall and a violation of the membrane stability [49]. The sizes of AuNPs in both types of heterostructures discussed seem to be nearly comparable: 6–9 nm for Au/M/CFR-PEEK and 9–11 nm for Au/M/Ti [24] (M = Ir, Pt, PtIr). At the same time, a higher surface concentration of gold was achieved in the current work: 10–11 μg/cm^2^ vs. 2–3 μg/cm^2^ in [24]. This could also be the cause of the observed bactericidal effect, because antibacterial properties are proportional to the gold concentration [50]. Thus, these results may be a primary basis for the lower limit values for determining the effective content of AuNPs obtained by the MOCVD method on the noble metal coatings. However, a clearer comparison is not yet possible due to the different coated carriers and research methods.

### 3.8. Histological Studies

#### 3.8.1. Heterostructures on CCM

Morphological study of the sample groups of pristine and coated CCMs discs did not show a noticeable difference between the parameters for the samples after 1 and 3 months of subcutaneous implantation in rats. This may be due to the specific filamentous structure of such discs, which caused the samples to sprout with host‘s fibrous capsule (FC) fibers. Selected histological findings for samples after 1 month of implantation are summarized in Table 3.

**Figure 13:**
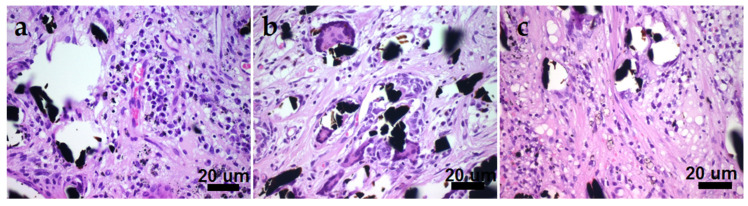
CCM sample micrographs demonstrating a large number of FBCs, macrophages, and lymphocytic cells ((**a)**—Pt/CCM sample, (**b**)—Ag/CCM sample, (**c**)—Au/Pt/CCM sample). The black inclusions are carbon fibers.

On the outside of all the samples, FCs were formed by classic loose connective tissues and structurally identical in terms of relative location and the thickness of individual fibers. Inside the samples, FC fibers formed a cell-filled fibrous base with a predominance of collagen. The host organism formed an insulating capsule around each fiber. In addition to the cells usual for loose connective tissue, macrophages, “foreign body” cells (FBCs) and lymphocytes were present in all the studied samples (Table 3). The histological differences between the samples were in the number of cells of each type and the density of collagen fibers (Figure 13).

Ideally, all parameters listed in Table 3, with the exception of microvessels, should be kept to a minimum. This would reflect the minimal reaction to the implantation of a foreign body. 

For a pristine CCM sample, the inflammation process was quite intense, and the active phase had not yet ended. Note that the inflammation appeared to be caused by substances releasing from the fibers. This was indicated by an increase in the number of cells near the fibers (Figure 13). The appearance of different types of cells indicated both a nonspecific immune response and an antigenic one, and their non-decreasing number of lymphocytes showed that the implanted sample continued to constantly supply antigenic determinants. The application of both a continuous film (Pt) and nanoparticles (Ag) did not noticeably change the observed pattern. A similar trend was noted for in vitro cytotoxicity (Section 3.6).

The Au/Pt/CCM sample group showed the least signs of inflammatory reaction (Table 3, Figure 13). Thus, the application of AuNPs seemed to level the observed negative effects and contributed to an increase in the number of microvessels. This observation correlates with biological in vivo benefits of AuNPs. For example, the increased vascularization and stimulated regenerative processes were recently shown for human culture of fibroblasts combined with AuNPs in the treatment of experimental burns in rats [51]. In our case, it can be assumed that the immune system initially reacted actively to the toxicant released by CCM, and then the immune response decreased due to the direct immunosuppressive effect of gold [52]. 

The observed “positive” in vivo result for Au/Pt/CCM, compared to the “negative” in vitro result (Section 3.6), may give priority to the hypothesis that the toxicant eluted by CCM reduces mitochondrial activity of cells to exhibit a direct cytotoxic effect. However, the methodology of these experimental studies did not allow direct correlations between in vitro and in vivo results. In fact, an extract from the sample was used in the cytotoxicity testing, i.e., the amount of toxicant was limited, and it could be completely inactivated due to interaction with cells. During implantation, the toxicant was released constantly. Therefore, there was a constant activation of nonspecific immunity. This shows the importance of preliminary in vivo studies to assess the biological potential of new biomaterials.

#### 3.8.2. Heterostructures on CFR-PEEK

**Figure 14:**
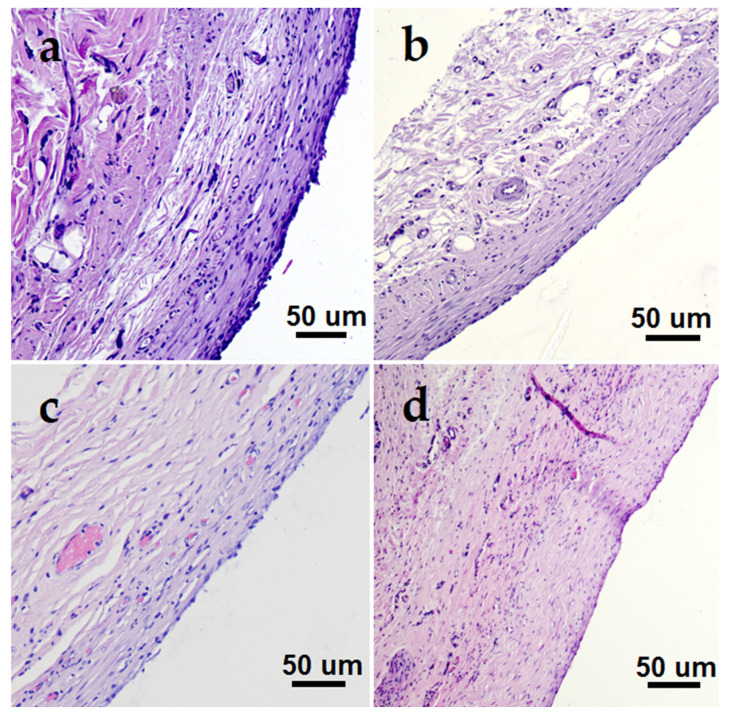
Fibrous capsule micrographs demonstrating different morphology structures ((**a**)—dense capsule around CFR-PEEK, (**b**)—two-layered capsule around Ag/PtIr/CFR-PEEK, (**c**)—microvessel-enriched capsule around Au/CFR-PEEK, (**d**)—capsule of maximum thickness within a studied series around Au/Pt/CFR-PEEK).

The samples on CFR-PEEK carriers generally showed better biocompatibility after 1 month subcutaneous implantation in rats than those on CCM. The scoring of histological indicators is presented in Table 4. The examples of fibrous capsule microphotographs are shown in Figure 14. In general, the inflammation process in the case of pristine CFR-PEEK samples was less pronounced than for the previously studied Ti alloy [24]. The application of noble metal-based heterostructures further improved the in vivo biocompatibility of the polymer material in terms of a number of important indicators.

Note that the Au/CFR-PEEK sample group was the only one characterized by a diffuse arrangement of fibroblasts in the thickness of the entire FC, in greater numbers than fibrocytes. The continued activity of the process may be related to the smallest roughness of the sample and the smallest size of gold particles with particular bioactivity [53,54]. The nonspecific suppressive and toxic effect of AuNPs was most pronounced on non-dividing cells (such as fibrocytes) or slowly dividing cells. While actively dividing, fibroblasts were apparently able to overcome the suppressive action of AuNPs. In the remaining cases, an equal ratio of fibroblasts and fibrocytes was observed, indicating normalization of the fibrosis process.

**Figure 15:**
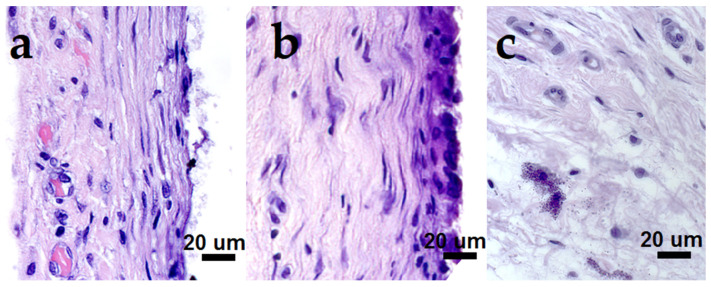
Features of the fibrous capsule cellular content surrounding the implanted materials, bar = 20 µm ((**a**)—capillaries, fibroblasts around Au/PtIr/CFR-PEEK, (**b**)—fibrocytes in the hyperchromic band and lymphocytic infiltration around CFR-PEEK, (**c**)—mast cells in the outer capsule layer around Ag/Ir/CFR-PEEK).

Most of the coated samples showed lower tissue lymphocytic infiltration compared to the pristine CFR-PEEK disc (Table 4, Figure 15). This confirmed a good bio-inertness of the obtained materials. The FCs around the samples were thin, uniform in thickness from all sides, and mainly consisted of collagen fibers. Further, we will discuss the samples sequentially in terms of various FC parameters.

Since mast cells (Table 4) were removed from the fibrous capsule during implant engraftment, we could use this parameter to estimate the time characteristics of this process. Herein, AgNP heterostructures, for which mast cells were still present inside the FC or in large amounts on the outer side of the FC, were characterized by a noticeably later completion of this phase. To a lesser extent, this could also be noted for the samples containing Ir films. 

Regarding the structure, a single-layer FC is desirable. Both a pristine CFR-PEEK carrier and most of the coated samples met this requirement. However, the PtIr/CFR-PEEK, Au/PtIr/CFR-PEEK, and Ag/PtIr/CFR-PEEK samples containing a bimetallic continuous film clearly exhibited double-layer FCs (Figure 14b). This result correlates with that obtained earlier in the study of similar film heterostructures on a metal carrier (Ti discs) [24]. There was a pronounced increase in FC in the interval of 1 and 3 months of implantation probably due to the slow release of platinum from the galvanic pair. In the current study, we could also assume that the formation of a distinct second layer of FC was associated with the accumulation of this new toxic agent, which was different from the rest of the samples. Thus, the use of a bimetallic PtIr coating as a continuous “bottom” layer for film heterostructures led to a decrease in biocompatibility. The monometallic film-base avoided such problem.

Regarding fiber packaging (Table 4), the least dense FC was optimal. For a series of samples with noble metal coatings (Pt, Ir, PtIr), FC was comparable in this parameter with pristine CFR-PEEK, i.e., relatively dense (Table 4, Figure 14a). The Ir-coated sample may represent a minimum value here, probably related to maximum roughness and minimum particle size on the surface or the specific effect of this noble metal. The further surface modification with AgNPs generally did not change the picture. The deposition of AuNPs (both on PEEK and on noble metal surfaces) apparently led to a slight improvement in the considered parameter. In general, the series of Au-containing samples (Au/CFR-PEEK and Au/M/CFR-PEEK, M = Ir, Pt, PtIr, Figure 14c,d) exhibited a lower FC density: moderate (2 scores) vs. severe-moderate (3–2 scores).

Regarding a hyperchromic stripe (Table 4), the minimal thickness reflected the minimal activity of fibrous cells (fibroblasts and fibrocytes). However, in the case of the pristine CFR-PEEK samples, this indicator was the most pronounced (3 scores). The presence of nanoparticles improved the discussed characteristic. This effect of the obtained AuNPs was superior to that of AgNPs: 0–1 scores over a series of Au/CFR-PEEK and Au/M/CFR-PEEK vs. 1–2 scores over a series of Ag/M/CFR-PEEK (M = Ir, Pt, PtIr).

Regarding microvessels (Table 4), their maximum amount in FC was desirable. In fact, the formation of microvessels inside the FC reflected the end of the engraftment process of the implanted object and its consolidation with the body’s own structures, because the processes for the delivery of oxygen and nutrients were launched [55]. Note that microvessels in the periphery (on the outside of the FC) were not such an indicator, because they were necessary for the nutrition of fibroblasts and other cells involved in the synthesis and organization of the FC around the implant. Therefore, without considering the external microvessels, one can reach clear conclusions on the positive effect of gold (Table 4). In particular, the corresponding parameter was 2 scores for Au/CFR-PEEK, while all the series of the Au/M/CFR-PEEK (M = Ir, Pt, PtIr) group were characterized by non-zero parameters. The promotion of AuNPs for microvessel formation was noted above for the Au/Pt/CCM sample group (Table 3, Section 3.8.1). The most promising here was the Au/Ir/CFR-PEEK sample with the maximum parameter value (3 scores).

In summary, the comparative analysis showed that the implanted AgNP heterostructures were characterized by acceptable biocompatibility in vivo, being better than for pristine CFR-PEEK. However, AuNP heterostructures showed the overall best histological characteristics. The developed surface morphology of the “bottom” layer of the heterostructure, i.e., a continuous noble metal film, also favored the biocompatibility of the implanted material. Contrarily, the bimetallic composition of this film led to undesirable specific reactions.

## 4. Conclusions

Following recent promising biological results exhibited by noble metal films combined with Ag or Au nano ensembles on titanium implant materials, here we applied such heterostructures to modify the surface of two types of carbon-based materials: carbon composite fiber material (CCM) and carbon-fiber-reinforced polyetheretherketone (CFR-PEEK).

For the formation of the “bottom” layers of heterostructures, namely, Pt, Ir of PtIr films with a developed morphology, we applied the MOCVD method with specially selected volatile precursors. Importantly, the application of Pt or Ir layer with a thickness ≥ 1 μm provided X-ray density ≥ 500 HU/cm^2^ for the samples on CFR-PEEK, while a pristine carrier was characterized by ~200 HU/cm^2^ for 1–2.5 mm thick discs. Near linear dependences of X-ray density on the noble metal film thickness are promising to adjust the required contrast of the carbon implant images on tomographs.

As “top” antibacterial components of heterostructures, AuNPs (10–11 μg/cm^2^) and AgNPs (4–6 μg/cm^2^) were formed on the surface of pristine and noble-metal-coated implant materials through pulse-MOCVD and PVD, respectively. Under the same conditions, MOCVD allowed obtaining conformal isolated AuNPs on the CFR-PEEK-based samples (3–9 nm), but nanoclusters of up to 45–60 nm on the CCM samples. The PVD method allows reducing the particle size difference on pristine carriers: AgNPs of 18–26 and 13–20 nm were deposited for CCM and CFR-PEEK, respectively. However, on the surface of noble metal films, AgNPs are coalesced into clusters with sizes reaching 60 nm.

In vitro studies showed high biocompatibility of all CFR-PEEK-based samples, but an intrinsic cytotoxicity of the pristine CCM, which did not decrease when film structures were applied. In vivo histological studies after subcutaneous implantation in rats confirmed the difference in the characteristics of fibrous capsules due to the different shapes of the implanted carrier discs: filamentous CCM and dense CFR-PEEK. However, in both cases, the application of AuNP heterostructures noticeably improved histological indicators. For a series of CFR-PEEK-based samples, it was shown that the use of AgNP heterostructures also improved biocompatibility in vivo compared to uncoated material, but the effect was not as pronounced as that for AuNPs. 

In vivo studies of antibacterial properties showed complete inhibition of the growth of *S. aureus*, *S. epidermidis*, *P. aeruginosa*, *Str. pyogenes* and *Ent. faecium* colonies for all the AgNP heterostructures. The obtained AuNP heterostructures also exhibited noticeable antibacterial action, causing strongly stunted growth. 

These results underline the application potential of precious metal-containing film heterostructures for improving the characteristics of carbon-based materials for implantation. Finding the desired balance between high in vivo biocompatibility and improved antibacterial properties of the material may be a direction for further research.

## Figures and Tables

**Table 1:** Deposition conditions of the noble metal films and the sample designations. The samples Ir/CCM, Ir/CFR-PEEK, Pt/CCM, Pt/CFR-PEEK and PtIr/CFR-PEEK (without/with surface modification by Ag and Au) were selected for biological studies.

Film	Carrier	Deposition Conditions						Time (h) *	Samples
		Precursor	Sample Mass (mg)	Temperature (°C)		Gas Flow Gas (L/h)			
				Evaporator (*p* Prec._._, Torr [25])	Deposition	Carrier (Ar)	Reagent (O_2_)		
Ir	CCM	Ir(acac)(CO)_2_	60	70 (0.02)	280	2	2	2	Ir/CCM
	CFR-PEEK		80	83 (0.07)	320			4	Ir/CFR-PEEK
			60					2	Ir/CFR-PEEK-2
			200					8	Ir/CFR-PEEK-8
			300					12	Ir/CFR-PEEK-12
Pt	CCM	(CH_3_)_3_Pt(acac)Py	80	70 (0.06)	280	2	1	2	Pt/CCM
	CFR-PEEK		100	115 (0.15)	320			4	Pt/CFR-PEEK
			80					2	Pt/CFR-PEEK-2
			200					6	Pt/CFR-PEEK-6
			350					12	Pt/CFR-PEEK-12
PtIr	CFR-PEEK	(CH_3_)_3_Pt(acac)Pyand Ir(acac)(CO)_2_	65 and 65	105 and 80 (0.06 and 0.06)	310	2	1.5	2	PtIr/CFR-PEEK
			130 and 130					4	PtIr/CFR-PEEK-4

* The deposition time is indicated for one side of the disc. For biological research, the samples were covered on both sides.

**Table 2:** X-ray density values of the series of Ir/CFR-PEEK and Pt/CFR-PEEK samples in comparison with CFR-PEEK discs.

Sample	Geometric Area (cm^2^)	Coating Thicknesses (μm)	X-ray Density * (HU/cm^2^)
CFR-PEEK disc	3.14 ± 0.08 7.06 ± 0.08	-	184 ± 4 200 ± 4
Ir/CFR-PEEK-2	7.06 ± 0.08	0.80 ± 0.04	324 ± 6
	3.14 ± 0.08	0.50 ± 0.03	217 ± 4
Ir/CFR-PEEK-8	3.14 ± 0.08	1.80 ± 0.09	865 ± 17
Ir/CFR-PEEK-12	3.14 ± 0.08	2.20 ± 0.11	1044 ± 21
Pt/CFR-PEEK	7.06 ± 0.08	1.40 ± 0.07	836 ± 17
Pt/CFR-PEEK-6	7.06 ± 0.08	1.70 ± 0.09	916 ± 18
Pt/CFR-PEEK-12	7.06 ± 0.08	2.50 ± 0.13	1162 ± 23

* Hounsfield scale (X-ray density of distilled water = 0 HU (densitometric values).

**Table 3:** Scoring of histological features of pristine and coated CCM samples after subcutaneous implantation in rats for 1 month.

Samples	Fiber Packaging	Microvessels	Macrophages	Lymphocytes	FBCs *
CCM	2	0	3	2	1
Pt/CCM	3	1	2	3	3
Ag/CCM	1–2	1	1–2	2	2
Au/Pt/CCM	0–1	2	1	1–2	0–1

* FBCs = “foreign body” cells.

**Table 4:** Scoring of histological features of pristine and coated CFR-PEEK samples after subcutaneous implantation in rats for 1 month.

Sample	Fiber Packaging	Hyperchromic Stripe	Microvessels	Lymphocytes	Mast Cells
CFR-PEEK	3	3	0	2	0
Au/CFR-PEEK	2	0	2	1	0
Pt/CFR-PEEK	3	0/1	2	3	0
PtIr/CFR-PEEK	3	3	1	0	0
Ir/CFR-PEEK	1	0	0	0	1
Au/Pt/CFR-PEEK	2	1	0/1	0	0
Au/PtIr/CFR-PEEK	2	0	1	0	0
Au/Ir/CFR-PEEK	2	0	3	1	2 *
Ag/Pt/CFR-PEEK	3	2	1 *	0	1
Ag/PtIr/CFR-PEEK	2	1	2 *	0	1
Ag/Ir/CFR-PEEK	3	2	0	0	3 *

* Observed on the outer side of the FC.

## Data Availability

The data presented in this study are available in this article or on request from the corresponding author.

## Supplementary Materials

The following supporting information can be downloaded at: https://www.mdpi.com/article/10.3390/biomedicines10092230/s1, Figure S1: XPS spectra of Ir/CCM with fitting of O1s (a), C1s (b); Figure S2: XPS spectra of Pt/CFR-PEEK with fitting of C1s (a), O1s (b).
