# Supplementary material for: Biological Studies of New Implant Materials Based on Carbon and Polymer Carriers with Film Heterostructures Containing Noble Metals
Source: Biomedicines. 2022 Sep 8;10(9):2230. doi: 10.3390/biomedicines10092230 (PMC9496383; doi:10.3390/biomedicines10092230)
Supplement: Supplementary file 1 [file biomedicines-10-02230-s001.zip › biomedicines-1886573-supplementary.pdf]

Biological studies of new implant materials based on carbon and polymer carriers with film heterostructures containing noble metals

Svetlana I. Dorovskikh[1], Evgeniia S. Vikulova[1], David S. Sergeevichev[2], Tatyana Ya. Gusel'nikova[1], Alexander A. Zheravin[2], Dmitriy A. Nasimov[3], Anatoliy I. Saprykin[1], Tamara V. Basova[1], Elena V. Chepeleva[2], Maria B. Vasilieva[2,4], Natalya B. Morozova[1,*]

1 Nikolaev Institute of Inorganic Chemistry SB RAS, 3 Lavrentiev Ave., Novosibirsk, 630090, Russia
2 Meshalkin National medical research center» of the Ministry of Health of the Russian Federation, 15 Rechkunovskaya Str., Novosibirsk, 630055, Russia
3 Rzhanov Institute of Semiconductor Physics SB RAS, 13 Lavrentiev Ave., Novosibirsk, 630090, Russia
4 Zelman Institute for the Medicine and Psychology, Novosibirsk State University, 1, Pirogov Str., 630090 Novosibirsk, Russia
* Correspondence: mor@niic.nsc.ru; Tel.: +73833309556

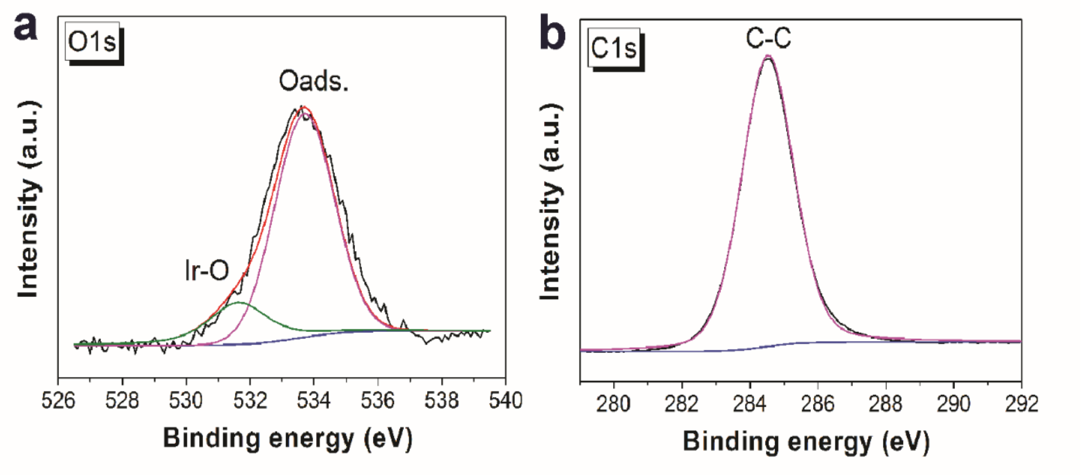

Figure S1. XPS spectra of Ir/CCM with fitting of O1s (a), C1s (b)

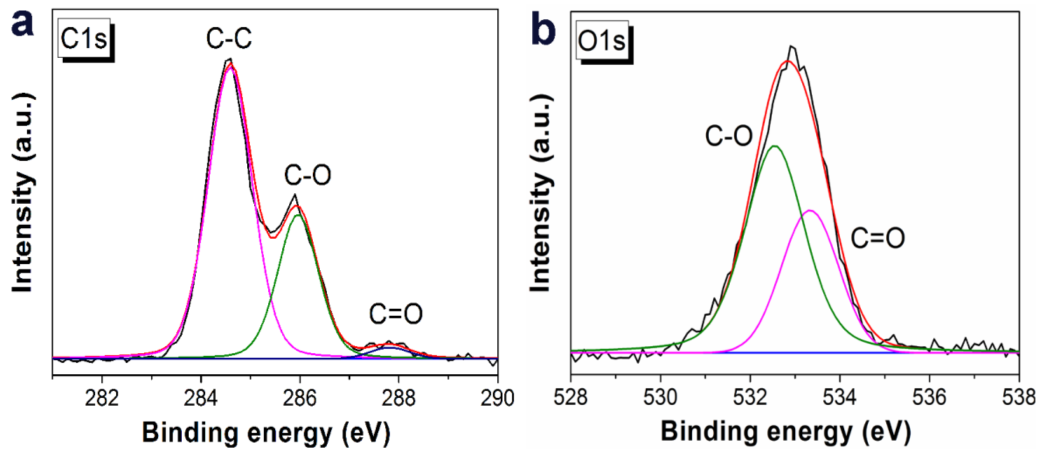

Figure S2. XPS spectra of Pt/CFR-PEEK with fitting of C1s (a), O1s (b)
